# Influencing factors of nursing discipline construction in a Chinese tertiary hospital: a qualitative study

**DOI:** 10.3389/fmed.2026.1796909

**Published:** 2026-07-14

**Authors:** Xuan Lu, Huanhuan Zhu, Min Li

**Affiliations:** 1 Department of Geriatrics, Nanjing Drum Tower Hospital, Affiliated Nanjing University Medical School, Nanjing, China; 2 Department of Nursing, Nanjing Drum Tower Hospital, Affiliated Nanjing University Medical School, Nanjing, China

**Keywords:** barrier, discipline construction, factor, nursing, qualitative

## Abstract

**Background:**

As a first-level discipline, nursing plays an increasingly important role in China’s healthcare system, and its sustainable development has become a key focus of current nursing research. The existing research on nursing discipline development is mostly quantitative analysis and lacks depth, failing to fully explore the experiences of nursing managers in practice and the influencing factors. This study adopted a descriptive phenomenological qualitative research design to explore the obstacles and promoting factors in nursing discipline construction and used Herzberg’s Two-Factor Theory as the theoretical basis for the research design and result interpretation. The research results can provide multi-dimensional references for policy formulation, talent cultivation, and clinical discipline construction, thereby promoting the high-quality and sustainable nursing discipline development.

**Objective:**

This study aimed to (1) understand the perceptions of nursing managers regarding the construction of nursing discipline, (2) assess barriers for nursing administrators in implementing the discipline of nursing on the unit in China, (3) explore how to promote the rapid and sustainable nursing discipline development in China. The study aims to provide effective theoretical support and practical guidance for future nursing discipline development.

**Methods:**

A descriptive phenomenological qualitative design was adopted. Purposive sampling was used to recruit nursing administrators receiving standardized discipline construction training from a tertiary hospital in Nanjing. Semi-structured interviews were implemented for data collection. Colaizzi’s analytical approach was used for qualitative data coding and analysis, and Herzberg’s two-factor theory guided the interview outline formulation and subsequent theme classification into hygiene and motivating factors.

**Results:**

A total of 18 nursing managers were interviewed in this study. This study coded and categorized qualitative data. Three thematic clusters were formed, covering eight subthemes in total: Hygiene factors: (1) uneven development of subspecialties, (2) irrational allocation of human resources, (3) lack of specialized nursing talent, (4) deficiencies in nurses’ scientific research competency, (5) lack of sustainability in development pathways, and (6) limitations in multispecialty integration. Adverse consequences of deficient hygiene conditions: (1) low subjective initiative, and (2) low engagement in scientific research and discipline construction. Motivators: No effective motivational factors were extracted from interview data. All eight subthemes constitute critical influencing factors restricting the construction and sustainable advancement of the nursing discipline.

**Conclusion:**

Semistructured interviews were conducted to explore the influencing factors and barriers to nursing discipline construction for healthcare professionals. While nursing discipline construction in China holds promising prospects, numerous issues remain to be addressed. A thorough understanding of the difficulties and obstacles in nursing discipline construction helped managers adopt targeted measures and advance the sustainable nursing discipline development.

## Introduction

### Background

Discipline construction is the foundation and core project of hospital construction, and with the development of modern society, as a first-level discipline, nursing discipline has occupied an increasingly important position in medical and health care and the overall construction of hospitals in China (1). As global health goals continue to advance, the demand for nursing services has increased, and the professional value of the nursing discipline is being increasingly recognized and appreciated (2). The Lancet published a special commentary titled “2020: Unleashing the Enormous Potential of Nursing,” urging governments and global health systems to allocate more resources and support to nurses and midwives to fully realize the professional value of nursing and unlock its vast potential (3). However, compared to other disciplines, nursing began relatively late in China, and there remain gaps and deficiencies in its construction. China’s 14th Five-Year Plan (2021–2025) and the 2035 Vision Outline emphasize the importance of respecting knowledge, valuing talent, and fostering innovation. They call for deepening reforms in talent development systems and mechanisms, comprehensively training, attracting, and effectively utilizing talent, and fully leveraging talent as the primary resource (4). The “National Nursing Development Plan (2021–2025)” advocates for nursing development to focus on meeting the clinical nursing needs of major diseases and key populations, strengthening nursing discipline construction, and promoting the training of nursing professionals and enhancement of nursing service capabilities through discipline development (5).

Despite the introduction of a number of supportive policies by the government, including the “National Nursing Development Plan” and the elevation of nursing to a first-level discipline, the professional innovation and development at the institutional level still face numerous bottlenecks. This study does not focus on the general constraints on nursing discipline development nationwide but rather focuses on the actual difficulties observed within a specific tertiary hospital. At the institutional level, discipline construction is hindered by multiple practical problems, including a shortage of senior nursing talents, an incomplete knowledge architecture of subsystems, and the personnel pressure brought about by population aging (6, 7). Currently, research on the nursing discipline development in China mainly focuses on establishing evaluation indicators and corresponding intervention strategies (6, 7), and most of the relevant evidence is obtained through quantitative survey designs, but there are still three obvious research gaps that have not been deeply explored: Firstly, a few existing studies have systematically classified and explained the restrictive factors for the development of the nursing profession using mature management theories (8, 9); Secondly, empirical evidence based on in-depth qualitative interviews with front-line nurse managers is extremely scarce (10, 11); Thirdly, previous studies rarely took the perspective of management practitioners to truly grasp the on-site obstacles in the construction of the local nursing profession (10). Given the above research deficiencies, it is urgent to take measures to deeply explore the internal factors that affect the construction of the nursing profession.

To better justify the theoretical framework of this study, Herzberg’s two-factor theory provides a classic and mature analytical perspective for exploring discipline development barriers. The two-factor theory was proposed by the renowned American psychologist Frederick Herzberg, based on Maslow’s hierarchy of human needs (11). The core logic of this theory divides the factors influencing the workplace into two mutually exclusive categories: hygiene factors and motivating factors. Hygiene factors refer to the objective environmental and resource conditions, including personnel allocation, disciplinary layout, research support conditions, and interdisciplinary collaboration resources, which are important fundamental guarantees for stable discipline construction. In contrast, motivating factors focus on intrinsic career incentives, such as professional recognition, academic achievements, and personal growth opportunities. These factors drive nurses to actively participate in discipline innovation and scientific research development (12).

This two-factor analysis framework is highly consistent with the current practical challenges in the construction of the nursing discipline: the discipline development is simultaneously constrained by insufficient external support resources as well as insufficient internal work motivation and participation enthusiasm (13). Moreover, intervention measures based on the two-factor theory have been widely applied in fields such as nursing management, education management, and human resource management, and have achieved positive results (8, 9, 14). Therefore, by applying Herzberg’s two-factor theory, it is possible to systematically classify, interpret, and integrate the various obstacles hindering the nursing discipline development, avoiding superficial phenomenological descriptions, and thus providing a solid theoretical basis for exploring the fundamental reasons for the development bottlenecks (11, 14). Accordingly, the study adopts this classic theory as its core analytical framework and employs semi-structured, in-depth interviews to comprehensively explore the lived experiences of nursing managers regarding nursing discipline development. The phenomenological methodology was chosen to capture the subjective realities of participants, while Herzberg’s framework provides a conceptual structure for interpreting and organizing the emerged themes. This integrated approach aims to deeply investigate the challenges and influencing factors related to discipline construction, providing a foundation for further promoting nursing discipline *development* and formulating individualized strategies.

## Methods

### Design and setting

From July 28 to August 31, 2025, a descriptive phenomenological approach, rooted in Husserl’s philosophical tradition and operationalized through Giorgi’s methodological framework, was used to examine the lived experiences of nursing managers in nursing discipline construction and to explore the confusion and influencing factors of their discipline construction at Nanjing Drum Tower Hospital, Affiliated with Nanjing University Medical School. Face-to-face, semi-structured, in-depth interviews were conducted with eligible nursing managers to collect information on their lived experiences and perceptions.

This large general hospital is located in Nanjing, Jiangsu Province, China. Founded in 1892, Nanjing Drum Tower Hospital is one of the earliest Western-style hospitals in China, with a rich history and cultural heritage. It has since developed into a large, comprehensive tertiary-level hospital offering a full range of disciplines, a strong faculty, advanced medical technology, and robust scientific research capabilities. The hospital’s medical and nursing care services are recognized as industry-leading. The hospital comprises three campuses: the Drum Tower headquarters, the Jiangbei campus, and the Southern campus. It has 3,800 beds, nearly 6,000 employees, and handles over 10,000 outpatient and emergency visits daily. For three consecutive years, and has been selected as a pilot hospital for high-quality development among public hospitals in China. While the hospital has made significant progress in discipline development over many years, the nursing discipline has only been established for about 5 years and still faces some challenges in its development. Although this study recruited participants from a single tertiary hospital and included only nursing managers, which may limit the generalizability of the findings. Nevertheless, the selected hospital is a comprehensive tertiary medical institution featuring complete nursing subspecialty settings, standardized discipline construction systems, and typical clinical nursing management characteristics consistent with most general tertiary hospitals in China. The participants represented multiple professional departments and possessed rich, discipline-related management experience, thereby ensuring the adequacy and authenticity of the interview information.

### Participants

From July 28 to August 31, 2025, a purposive sampling method was employed. Nursing managers from specialist departments previously selected as training targets for nursing discipline development in the hospital were recruited as study participants. The inclusion criteria were as follows: (1) individuals with at least 3 years of experience in nursing management; (2) those possessing extensive professional nursing experience and holding an intermediate or higher professional title (i.e., nurse-in-charge, associate professor of nursing, associate professor of nursing). The exclusion criteria were: (1) individuals who refused to be recorded; (2) those who withdrew during the interview. In this study, data saturation was assessed based on the emergence of repeated content and the absence of new themes. During semi-structured interviews, continuous comparative analysis was conducted concurrently with data collection. Data saturation was deemed achieved when no new categories, concepts, or unique viewpoints emerged from consecutive interview transcripts, and the extracted themes were sufficiently repeated and mutually verified across participants. Subsequent recruitment of additional participants was not pursued (13).

### Interview outline

According to the purpose of the study and the interview subjects, the interview outline was designed using semi-structured interviews. Members of the research team, after reviewing the literature and considering the three-year nursing discipline plans developed by each specialty, initially drafted the interview outline. This draft was then revised based on expert consultations. Following thorough discussions with one qualitative research expert (master’s supervisor, professor of nursing), one deputy director of the nursing department involved in nursing discipline developments (with over 20 years of clinical experience, master’s supervisor, professor of nursing), and one nurse (with more than 10 years of clinical experience, master’s degree, and nurse-in-charge), we conducted pre-interviews with five nursing administrators from two key nursing specialties. The interview outlines were subsequently revised according to expert feedback and the pre-interview results, resulting in the finalized interview outline. The interview questions were as follows: (1) Can you describe whether your department is engaged in nursing discipline construction? If so, please specify the aspects involved. (2) Have you encountered any problems or challenges during the nursing discipline construction process? Please specify. (3) What support or assistance do you require for nursing discipline construction? Please specify. (4) Do you have any other opinions or suggestions regarding the construction of nursing disciplines?

### Data collection

The research team prepared an interview outline in advance to clarify the theme and process. The interview was conducted in a guided format, with attention to maintaining consistency in the content of questions and responses. The interview was structured into three stages: ice-breaking introduction, in-depth discussion, and summary feedback. Each session was limited to a duration of 90 min. Prior to the interview, the time and venue were determined by the department head, and a ‘Do not disturb’ sign was placed at the site to minimize external interference. The interview was conducted by two researchers in a division of labor. The first interviewer was an independent nursing researcher with no administrative management position in the target hospital, who was responsible for formal one-on-one interviewing, interactive communication, and real-time verbal prompting. The project leader, who serves as the deputy director of the nursing department, acted as the second researcher and only participated in on-site observation, note recording, and post-interview discussion rather than directly conducting participant interviews. This dual-researcher division strictly reduced potential power hierarchy bias caused by administrative roles.

All researchers possess substantial experience in nursing education, clinical management, and scientific research, enabling them to accurately grasp the genuine perspectives of front-line managers. To avoid analysis bias stemming from past experiences, the study adhered to a standard process. Data coding and theme extraction were completed independently by two individuals. Discrepancies were resolved through expert discussion and verified by team members to ensure the authenticity and objectivity of the results. Researchers continuously reflected on their own biases, striving to mitigate individual subjective tendencies and truly restore the actual views of the respondents.

### Data analysis

Data were collected and analyzed concurrently. Within 48 h of each interview, we employed researcher triangulation. Two researchers independently utilized NVivo 12 qualitative data analysis software to organize and analyze the audio recordings and written interview transcripts, followed by a comparison of their analytical results. In the event of any discrepancies, a third expert in the relevant field would jointly discuss the differences and reach a consensus. The data were analyzed using Colaizzi’s seven-step phenomenological analysis method (15). As a systematic analytical approach specifically developed for phenomenological inquiry, Colaizzi’s method encompasses the following procedures: (1) carefully reading all data to achieve immersion; (2) extracting significant statements pertinent to the investigated phenomenon; (3) formulating meanings from the significant statements; (4) grouping formulated meanings into clusters of themes through systematic thematic coding; (5) integrating themes into an exhaustive description of the phenomenon; (6) synthesizing the exhaustive description into the fundamental structure of the phenomenon; and (7) validating the findings through member checking. It should be emphasized that the thematic coding and categorization procedures employed in Step 4 were conducted within the phenomenological framework of Colaizzi’s method, serving as an embedded analytical technique to organize meaning units rather than constituting an independent thematic analysis methodology (e.g., Braun and Clarke’s reflexive thematic analysis). Herzberg’s two-factor theory was applied as the overarching theoretical framework to guide the development of the interview outline and to provide a conceptual structure for interpreting and organizing the emerged themes into hygiene factors and motivators, thereby ensuring theoretical coherence throughout the research process. To ensure the authenticity and accuracy of the research results, we employed the member checking method to verify the authenticity and accuracy of the extracted themes within 48 h. All participants (100% of the sample) were invited to review the preliminary thematic results. Participants were asked to confirm whether the summarized themes and descriptions were consistent with their actual experience and whether any key viewpoints were missing. Minor revisions were made based on participants’ feedback, including supplementing individual contextual descriptions of nurse burnout and optimizing the expression of interdisciplinary collaboration barriers. No core themes were revised or removed after verification, which fully confirmed the credibility and validity of the final findings.

### Ethical considerations

This study was approved by the Ethics Committee of Nanjing Drum Tower Hospital, Affiliated Hospital of Nanjing University Medical School (Approval No. 2021–523-02). All procedures in this study were strictly performed in accordance with the Consolidated Criteria for Reporting Qualitative Research (COREQ) to ensure standardized and transparent research reporting. Prior to the interview, all participants were provided with comprehensive study information, including confidential details. Participants were informed of their right to withdraw from the study at any time during the interview. They were also asked for their agreement to have the entire interview recorded. If participants declined recording, the interview was promptly switched to manual documentation. All participants provided signed written informed consent and participated voluntarily in this study.

## Results

### Participant characteristics

A total of 18 nursing managers were interviewed in this study. The participants’ ages ranged from 33 to 54 years (mean ± SD: 41.94 ± 5.51), and all were female. Their years of working experience ranged from 6 to 33 years (mean ± SD: 20.06 ± 7.24). Professional titles included six nurse-in-charge, 10 associate professors of nursing, and two professors of nursing. Their administrative positions comprised one Associate Director of Nursing and 17 Unit Nurse Managers. In terms of educational attainment, one participant possessed a doctoral degree, six held master’s degrees, and 11 had bachelor’s degrees. Besides, 10 participants were Clinical Nurse Specialists (CNS) and eight participants were Non-CNS. The general characteristics of the participants are presented in Table 1.

**Table 1:** General characteristics of participants.

Participant	Age (y)	Years of working experience	Professional title	Administrative position	Academic degree	Clinical Nurse Specialist
M1	46	26	Associate Professor of Nursing	Unit Nurse Manager	Bachelor’s Degree	Y
M2	54	31	Professor of Nursing	Unit Nurse Manager	Master’s Degrees	N
M3	48	29	Professor of Nursing	Unit Nurse Manager	Bachelor’s Degree	Y
M4	44	20	Associate Professor of Nursing	Unit Nurse Manager	Bachelor’s Degree	N
M5	33	6	Nurse-in-Charge	Unit Nurse Manager	Master’s Degree	N
M6	41	22	Associate Professor of Nursing	Unit Nurse Manager	Bachelor’s Degree	N
M7	51	33	Nurse-in-Charge	Unit Nurse Manager	Bachelor’s Degree	N
M8	39	16	Associate Professor of Nursing	Unit Nurse Manager	Master’s Degrees	Y
M9	40	14	Nurse-in-Charge	Unit Nurse Manager	Bachelor’s Degree	Y
M10	37	14	Nurse-in-Charge	Unit Nurse Manager	Master’s Degree	Y
M11	40	17	Associate Professor of Nursing	Unit Nurse Manager	Bachelor’s Degree	N
M12	46	26	Associate Professor of Nursing	Unit Nurse Manager	Bachelor’s Degree	Y
M13	40	17	Nurse-in-Charge	Unit Nurse Manager	Bachelor’s Degree	Y
M14	45	27	Associate Professor of Nursing	Unit Nurse Manager	Bachelor’s Degree	Y
M15	35	13	Associate Professor of Nursing	Unit Nurse Manager	Master’s Degrees	Y
M16	39	18	Nurse-in-Charge	Unit Nurse Manager	Bachelor’s Degree	N
M17	39	18	Associate Professor of Nursing	Unit Nurse Manager	Master’s Degrees	N
M18	38	14	Associate Professor of Nursing	Associate Director of Nursing	Doctoral Degree	Y

Y: Clinical Nurse Specialist, N: Non-Clinical Nurse Specialist.

### Themes and subthemes

Guided by Herzberg’s two-factor theory, this study coded and categorized qualitative data. Three thematic clusters were formed, covering eight subthemes in total: Hygiene factors: (1) uneven development of subspecialties, (2) irrational allocation of human resources, (3) lack of specialized nursing talent, (4) deficiencies in nurses’ scientific research competency, (5) lack of sustainability in development pathways, and (6) limitations in multispecialty integration. Adverse consequences of deficient hygiene conditions: (1) low subjective initiative, and (2) low engagement in scientific research and discipline construction. Motivators: No effective motivational factors were extracted from interview data.

#### Theme 1: hygiene factors

##### Uneven development of subspecialties

The establishment of subspecialties can advance the specialization and refinement of disciplines, which is crucial for promoting their high-quality development. However, excessive proliferation of nursing subspecialties may result in imbalanced growth, hindering the formation of distinct disciplinary characteristics and the core competitiveness of each subspecialty.

*“The subspecialty within my department is currently divided into six nursing subspecialties, each focusing on different areas. Each sub-specialty is responsible for two or three staff members. Over the past few years, some subspecialties have developed significantly, establishing specialized nursing practices and key technologies within the department. Among these, two subspecialties are clearly leading at the national level in China. However, the development of certain other subspecialties has remained nearly stagnant, yielding no notable results.*” (M1).

*“The development of the five subspecialties in our department is uneven. While other specialties have progressed adequately, thyroid nursing remains underdeveloped. No specialized nursing techniques have been established, so patient care is limited to the routine practices of the department.” (frowns)* (M2).

##### Irrational allocation of human resources

The construction of the nursing field should take patient safety as the fundamental principle. Only by ensuring sufficient and reasonable human resources and ensuring the quality of clinical nursing can we steadily promote the discipline development.

*“The number of nursing staff in the department is insufficient, with several nurses currently on sick leave or maternity leave. It is very challenging for nurses to work night shifts, and there is limited availability of manpower and time to conduct scientific research. Consequently, the discipline development and construction are also constrained.”* (M4).

*“The surgical anesthesia department handles a high volume of operations. In recent years, the hospital has been continuously expanding, requiring nurses to be deployed to support the branch. Many nurses often cannot leave work on time and must use their rest periods to gather at the hospital and work overtime to plan the development of the department.” (sigh)* (M3).

*“The unreasonable structure of human resources has long been a significant obstacle in the development of our Orthopedics department. The number of nurses is insufficient, and many are very young, with relatively low levels of experience and professional titles. Although young nurses bring enthusiasm, their ability to keep pace presents challenges in advancing the specialty.”* (M5).

##### Lack of specialized nursing talent

High-quality specialty construction depends on a skilled and dedicated talent team. As the talent gap in nursing discipline construction widens and the demand for qualified professionals increases, the urgency of talent development becomes more pronounced.

*“The training of specialist nurses in our department is a bottleneck. Due to the limited number of hospital training slots, our department frequently applies for overseas nurses to participate in the national or Jiangsu Province diabetes specialist nurse training programs. However, after multiple rounds of screening by both our department and the nursing department, no training places are allocated to us. The shortage of specialist nurses is hindering our discipline development.”* (M1).

*“The nurses in our department possess a limited level of specialized knowledge and only a partial understanding of the discipline development. For example, it has been demonstrated that appropriate techniques in Chinese medicine benefit mothers who are separated from their infants during breastfeeding. However, the nurses often have only superficial knowledge of Chinese medicine and lack professional operational skills. Therefore, it is urgent to train relevant specialized personnel.”* (M6).

*“Our department is weak in personnel training, and many colleagues, especially nurses, need to strengthen their theoretical knowledge and practical skills. Due to the insufficient level of specialized knowledge, whenever a subspecialty development task is assigned, it cannot be effectively implemented, and no one is able to carry it out. As a result, the development of specialties in our department is relatively underdeveloped, making it difficult to overcome the current challenges.”* (M7).

##### Deficiencies in nurses’ scientific research competency

Discipline construction should be supported by robust scientific research; however, insufficient research capacity results in low-quality output, making it difficult to overcome research bottlenecks and impeding the high-quality advancement of the discipline.

*“I found that scientific research is a significant shortcoming of our department because there are still very few personnel with strong research capabilities. Most staff members require further training. Even among nursing graduates, research abilities* var*y; professional nursing master’s graduates and academic nursing master’s graduates differ in this regard. Therefore, their roles in the discipline development are not the same.”* (M10).

*“As the nnit nurse manager, Nursing graduate students are the primary contributors to departmental scientific research output. Nevertheless, our departments currently have no graduate-prepared nurses, and most frontline nurses are reluctant to invest spare time in pursuing postgraduate studies. Both ourselves and our team exhibit limited research capacity. Although we have been actively encouraging staff to take postgraduate entrance examinations and are also striving for further academic improvement themselves, the persistent lack of research talents forms a vicious cycle. Insufficient team research capacity ultimately creates substantial barriers to sustainable nursing discipline development” (M12, M16).*

*“Although nurses in the key anesthesiology and surgical nursing disciplines have abundant opportunities for domestic academic exchanges, they lack access to international academic communication platforms. High English proficiency requirements for international academic activities cannot be met due to nurses’ overall limited English competence, which hinders external academic communication and learning. Furthermore, many nurses show enthusiasm for scientific research writing but lack fundamental research, including basic capabilities in literature retrieval and reading. Their inadequate English level further exacerbates these deficiencies and makes targeted professional guidance difficult to implement efficiently. Collectively, these shortcomings greatly limit the generation of innovative professional achievements and substantially constrain the high-quality nursing discipline development.” (M11, M18).*

*“Our department (Oncology Department) currently has a nursing graduate student. This year, I have been implementing the ‘old to new’ teaching model. After 1 year of implementation, I found that the effect is not significant. The scientific research quality and innovation ability of junior college students or undergraduates who have not been trained by graduate students remain somewhat lacking. As a result, this approach has limited impact on promoting the discipline development*.” (M17).

##### Lack of sustainability in development pathways

The sustainability of the discipline depends on adhering to the principles of sustainable development and dynamically analyzing the status of China’s nursing discipline in the global context, along with its strengths and weaknesses. This approach facilitates enhancing the discipline’s capacity for sustainable growth and promotes the construction and advancement of the nursing field.

*“Although the sustainability of discipline development is crucial for the growth of the specialty, the sustainability of our department’s discipline development is relatively low. For example, some nurses do not continue to pursue research topics after the initial application of a research project. They may only submit the project proposal without implementing it, resulting in no scientific research output. This lack of continuity in discipline development is very concerning to me.”* (M2).

*“Every year, the department formulates a specific development plan for discipline construction. Most nurses have corresponding goals and plans, but during implementation, few are able to adhere to them. As a result, much of the specialty nursing work remains stagnant, which makes me feel very worried, overwhelmed, and helpless.” (shrugs) (M8).*

##### Limitations of multispecialty integration

Nursing discipline development requires breaking through disciplinary barriers, fostering communication and collaboration between nursing and other fields, and establishing a strong foundation to promote the cross-integration of nursing disciplines.

*“In clinical practice, we found that appropriate Chinese medicine techniques are particularly easy to implement and are well-liked by patients. From a social and economic perspective, these methods are also highly beneficial. The positive effects are evident in the clinical management of maternal pain and milk stasis in obstetrics and gynecology. However, our department lacks a professional Chinese medicine specialist, which hinders the nursing staff from effectively carrying out these interventions. As a result, non-pharmacological measures cannot be used to adequately relieve postpartum discomfort in patients.”* (M14).

*“Most patients in the geriatric department have multiple coexisting diseases. It is challenging for specialist doctors or nurses to provide effective treatment and care for elderly patients. However, our department still lacks a standardized, long-term cooperative medical team. Additionally, rehabilitators, dietitians, specialist nurses, and other support staff are relatively scarce, necessitating increased support from the hospital.”* (M13).

#### Theme 2: adverse consequences of deficient hygiene conditions

##### Low subjective initiative

Growing demand for healthcare services imposes sustained heavy workloads on clinical nurses. Such unfavorable working conditions, categorized as inadequate hygiene conditions, negatively dampen nurses’ work motivation, hinder personal career development, and further constrain the progress of specialized nursing disciplines.

*“Development presents significant challenges for my department. A persistent trend has been observed: the enthusiasm of junior nurses is lower than that of more experienced colleagues. Senior nurses, often driven by pragmatism, recognize the substantial impact of professional titles on their entire careers and are therefore motivated to enhance their own skills and contribute to departmental discipline construction. In contrast, younger nurses (typically undergraduates) exhibit less engagement, perceiving departmental discipline development as irrelevant to them and showing little interest in pursuing professional title advancement. This disparity is deeply concerning.”* (M8).

##### Low engagement in scientific research and discipline construction

Apart from weakened individual initiative, the above-mentioned hygiene deficits including insufficient research competence and talent shortage further translate into practical obstacles to discipline development, manifested as low engagement in scientific research and discipline construction among nurses.

*“I observed that nurses in our department dedicate 8 h daily to clinical work, arriving and departing on schedule to ensure effective patient care. However, when discussing departmental discipline construction or scientific research development, they demonstrated limited awareness and disinterest. Initially, some nurses showed active participation, but over time, this engagement diminished progressively. Previously announced topics often failed to be implemented, and as time elapsed, motivation to address these areas waned further, ultimately resulting in inaction. I am uncertain how to effectively address this situation.”* (M9).

*“In clinical nursing, I found that it was not difficult for nurses to do anything within the clinical nursing work, and they were willing to do it, but if they were asked to do something related to scientific research or to do something for the development of the department, they would be very resistant, thinking that it had nothing to do with them, and they were particularly concerned about overtime work and had complaints.”* (M10).

## Discussion

Although nursing discipline construction has been promoted in Nanjing Gulou Hospital since 2017, due to the impact of the epidemic after three long years, the implementation time of this study was mainly in the months after the opening of the epidemic, the clinical medical staff seemed to remain immersed in COVID-19 prevention and control routines and a state of exhaustion, and has not yet adjusted to the work state. A variety of factors have contributed to the current undesirable state of discipline development. Nursing administrators are not satisfied with this status quo.

Methodologically, this study integrated descriptive phenomenology as the overarching research philosophy, Colaizzi’s seven-step method as the specific phenomenological data analysis technique, and Herzberg’s two-factor theory as the theoretical framework. Descriptive phenomenology, grounded in Husserl’s philosophical tradition and operationalized through Giorgi’s methodological guidelines, provided the epistemological foundation for exploring the lived experiences of nursing managers. Colaizzi’s systematic analytical procedures, particularly the thematic coding embedded within Step 4, enabled the rigorous extraction and categorization of meaning units from the interview transcripts. It is important to clarify that the thematic procedures employed in this study were integral to Colaizzi’s phenomenological method, not an independent thematic analysis approach. Herzberg’s two-factor theory was subsequently applied to organize the emerged themes conceptually, distinguishing between hygiene factors related to work environment and conditions, and motivational dynamics reflecting individual-level challenges that require systemic attention. This tripartite integration ensured philosophical coherence, analytical rigor, and theoretical groundedness throughout the research process.

The participants were drawn from a range of clinical areas, including endocrinology, respiratory, neurosurgery, gastrointestinal surgery, orthopedics, anesthesia and surgery, obstetrics and gynecology, geriatrics, and other specialist areas. All the participants had rich nursing and management experience in their related specialties, and had worked for more than 10 years. They were all promoting nursing discipline construction projects. All of them could clearly understand and analyze the development of specialties in their departments, realize the shortcomings of their own department development, and have certain ideas and plans for the future of discipline development.

This study found that the uneven development of subspecialties was an important influence on the effectiveness of nursing discipline construction, which is consistent with the findings of previous research (16). The excessive specialization of nursing subdisciplines has directly led to the imbalance in the various discipline development. The underlying mechanism can be explained from two aspects: On one hand, excessive specialization causes the limited number of highly educated and highly qualified nursing professionals to be dispersed across multiple subspecialties, weakening the core competitiveness and research output capacity of individual specialties (17). On the other hand, after the specialization, the business volume and influence of each specialty vary significantly, resulting in the concentration of resources (such as research funds, training opportunities) toward the dominant specialties, forming the Matthew effect where “the strong become stronger and the weak become weaker,” which is consistent with the organizational ecology theory of discipline development (18). The results of this study revealed that insufficient uneven allocation of human resources was the key bottleneck restricting the balanced of the nursing discipline. This finding is consistent with relevant research in the field of nursing management in China (19), that is, the structural imbalance in the allocation of nursing personnel is the fundamental cause of the uneven development of specialized nursing. Unlike most existing studies which mainly focus on the shortage of human resources, this study further points out from the perspective of managers that the dilution of human resources caused by excessive specialization of sub-specialties is the core mechanism leading to the development gap, providing empirical evidence for the subsequent targeted allocation of human resources (17, 19).

This study found that a lack of specialized nursing talent, and deficiencies in nurses’ scientific research competency were key factors restricting nursing discipline development, correspond to the core goal of the “National Nursing Development Plan (2021–2025)” which states “strengthen the construction of specialized nurses and enhance the nursing service capabilities” (5). The results of this study suggested that the current training model, especially the training system for the research capabilities of nurses with undergraduate and below educational qualifications, still has a gap compared to the policy requirement of “high-quality and innovative” nursing talents. This provides empirical feedback from the clinical front for the subsequent optimization of the training plan (5). It is worth noting that this study shows that heavy clinical work is the main obstacle to the development of nurses’ research capabilities. The mechanism lies in that the time and energy of nurses are fixed resources. When the clinical workload reaches saturation, nurses cannot allocate sufficient time for literature reading, data analysis, and paper writing. This ‘clinical priority’ work mode squeezes out the survival space for research, causing research work to become an ‘extra burden’ rather than an organic part of career development, and ultimately forming a vicious cycle of ‘clinical-research’ time conflict (10). Ultimately, specialist departments of hospitals should give full play to the specialist guidance of specialist nurses and the role of nursing postgraduates in scientific research innovation, so as to optimize the internal organizational operation of nursing disciplines and promote long-term discipline development under the guidance of national nursing policies.

Meanwhile, this study reveals that the development path of the nursing discipline remains unclear. There is a lack of systematic planning and a shortage of mechanisms for long-term sustainable development. Some departments still do not attach sufficient importance to the construction of the nursing discipline and fail to recognize its core role in promoting the sustainable development of the profession (20). Therefore, nursing managers should formulate targeted construction plans based on actual clinical situations to ensure their strong practical feasibility. Integrating discipline management with administrative management also helps to effectively support the implementation of related work.

This study identified prominent deficiencies in the multidisciplinary integration of nursing. Modern scientific development emphasizes both disciplinary refinement and interdisciplinary collaboration, which is crucial for enhancing academic and research competitiveness. International institutions have maturely promoted interdisciplinary development. As early as 2006, the University of Southern California issued interdisciplinary research guidelines and established specialized committees, cross-college faculty appointments, and integrated talent training systems (21). Harvard University has also launched interdisciplinary curricula to cultivate comprehensive talents adapting to integrated scientific trends (22). Increasing domestic evidence also confirms that multidisciplinary integration facilitates disciplinary innovation. Accordingly, nursing should actively explore synergies with other disciplines, carry out interdisciplinary research, consolidate its unique research advantages, and further promote the sustainable nursing discipline development (23, 24).

According to Herzberg’s Two-Factor Theory, this study divided the factors influencing the construction of the nursing discipline into hygiene factors and motivators (12). Among them, inadequate working conditions are the core hygiene factor, which leads to two main negative consequences: low subjective initiative, and low engagement in scientific research and discipline construction. Moreover, no effective motivators were found in the interview data.

The nurses’ enthusiasm for discipline construction is influenced by the promotion rules, performance assessment, and hospital support system. Currently, performance compensation is mainly linked to clinical workload, while contributions to research and discipline development carry very little weight (25, 26). This greatly reduces the nurses’ enthusiasm for participation. Moreover, the promotion policy sets strict requirements for academic achievements, but most nurses lack solid research capabilities, so they are unwilling to engage in related work. The lack of systematic scientific research training, mentor guidance, and targeted incentive measures at the hospital level also hinders nurses from converting their enthusiasm into practical actions. This phenomenon is consistent with Herzberg’s view that inadequate hygiene conditions can render incentive measures ineffective (12).

Since hygiene conditions help eliminate job dissatisfaction, poor basic conditions and imperfect institutional arrangements leave nurses in a passive working state. At the same time, the lack of effective incentive factors such as professional recognition and development opportunities leads to a lack of intrinsic motivation to increase long-term investment (27, 28). Overall, the current construction of the nursing discipline is affected by the deficiencies in basic guarantees and the imperfect incentive system. To solve this problem, managers should first optimize working conditions and improve institutional mechanisms to make up for the deficiencies in hygiene conditions. On this basis, it is necessary to establish scientific incentive policies and training systems to fully mobilize nurses’ enthusiasm for research and innovation in the discipline (29, 30).

### Strengths and limitations

The primary strength of our study lies in the use of qualitative methods to explore the real experiences, influencing factors, and coping suggestions of nursing managers during the process of nursing discipline construction in Chinese general hospitals. However, a major limitation is the small sample size, which is restricted to individuals from Nanjing, thereby limiting the generalizability of the results to the entire country. Additionally, this study only considered the perspectives of nursing managers regarding nursing discipline construction and did not include other relevant stakeholders, such as physicians and patients. In order to make up for the shortcomings of the existing research, the sample size can be expanded in the future to include medical institutions and different medical staff. To effectively improve the representativeness of the sample, analyze the driving and restricting factors of nursing development, and formulate practical nursing optimization strategies.

## Conclusion

The influencing factors or barriers of nursing discipline construction for healthcare professionals were explored through semistructured interviews. Currently, despite the promising prospects for nursing discipline construction in China, numerous issues still need to be addressed, such as uneven development of subspecialties, irrational allocation of human resources, lack of specialized nursing talent, and weak scientific research support. For nursing managers, it is necessary to go deep into the clinical practice scene, accurately explore the existing problems of discipline development, combine the characteristics of the department and personnel structure, and formulate a clear level and practical landing plan. Additionally, they should enhance mechanisms for talent cultivation, performance evaluation, and scientific research incentives. Policy makers, on the other hand, should strengthen top-level design, coordinate the long-term development strategy for the nursing discipline, improve relevant management systems and supportive policies, increase funding and investment in platform construction, and promote the deep integration of discipline management with administrative management. Through targeted actions by managers and institutional-level policy support, these combined efforts can overcome developmental barriers and facilitate the rapid, standardized, and sustainable high-quality nursing discipline development.

## Data Availability

The raw data supporting the conclusions of this article will be made available by the authors, without undue reservation.
